# Comparison of the Efficacy of Tramadol and Paroxetine in the Management of Premature Ejaculation

**DOI:** 10.7759/cureus.10725

**Published:** 2020-09-29

**Authors:** Muhammad Fazal Ur Rehman, Ali Imran Zaidi, Tanveer Ul haq, Shoaib Rafique, Farman Ali

**Affiliations:** 1 Urology, Bakhtawar Amin Medical and Dental College, Multan, PAK; 2 Urology, Multan Institute of Kidney Diseases, Multan, PAK; 3 Internal Medicine, District Headquarter Hospital, Khanewal, PAK

**Keywords:** tramadol, paroxetine, premature ejaculation, latency time.

## Abstract

Objective

The goal of this study was to compare the efficacy of tramadol and paroxetine in the treatment of primary premature ejaculation (PE).

Study design

This study was a randomized controlled trial performed in the outpatient department of Nishtar Hospital, Multan, from January 2017 to January 2018.

Methodology

One hundred six patients were diagnosed with PE and included in the study. The patients were categorized into two groups receiving either tramadol or paroxetine through a lottery randomization method. The main variables were baseline PE, baseline satisfaction after intercourse, baseline intravaginal ejaculatory latency time (IELT), ejaculation control, difficulty in ejaculation, and after-treatment satisfaction with sexual intercourse and IELT. We used IBM SPSS Statistics for Windows, Version 23.0 (Armonk, NY: IBM Corp.) for data analysis, and p≤0.05 was considered statistically significant.

Results

Ejaculation control, difficulty in ejaculation, and distress due to ejaculation in patients in the tramadol group was noted as 24.5%, 7.5%, and 7.5%, respectively. Ejaculation control, difficulty in ejaculation, and distress due to ejaculation in the paroxetine group was noted as 49.1%, 17%, and 24.5%, respectively. The differences were statistically significant within the groups at baseline and after treatment of PE (p<0.001).

Conclusion

Tramadol is an effective and useful drug as compared to paroxetine for the treatment of PE. Tramadol can be used as an alternative to other medications for the treatment of lifelong PE.

## Introduction

In mammalians, a trend has been observed with regards to ejaculating quickly for the purpose of reproducing with multiple partners in order to share genetic material. In *Homo sapiens*, having multiple sexual intercourses is not only limited to reproductive purposes but also has important effects on quality of life. As males reach puberty, they start to engage in sexual intercourse, which alters their ejaculation time and perceived ejaculation control with the passage of time. Therefore, premature ejaculation is (PE) should not be considered or defined as pathological [1].

Delay in ejaculation with every intercourse can lead to negative consequences such as frustration, distress, and avoidance of sexual contact [2]. Ejaculation within one minute of vaginal penetration in every intercourse since an individual's first sexual episode is considered lifelong PE, which leads to a reduction in latency time, and more often, PE occurs when ejaculation is acquired in three minutes or sooner [3].

The management of PE remains a challenge for clinicians, patients, and their partners. The prevalence of PE is around 20% to 40% worldwide, and its diagnosis is often complicated [4]. The definition of PE frequently relies on ill-defined subjective descriptions, and as little as 5% of individuals fulfill the definition of PE according to the International Society of Sexual Medicine (ISSM) criteria [5]. Determining PE represents a challenge in that PE can be found in a person with frequent sexual interaction and rapid ejaculation or a person with infrequent sexual interaction and a single episode of PE.

Psychological or relationship problems, sexual performance anxiety, hyperthyroidism, acute or chronic prostatitis, diabetes mellitus, hypertension, and high body mass index (BMI) are causes of acquired PE [6] However, lifelong PE is related to a complex interplay of central and peripheral dopaminergic, endocrinological, serotonergic, epigenetic, oxytocinergic, and genetic factors [7]. In the treatment of PE, tramadol and sildenafil are not recommended. However, paroxetine and dapoxetine (selective serotonin reuptake inhibitors [SSRIs]), are recommended by the ISSM guidelines [8,9]. Tramadol could improve intravaginal ejaculatory latency time (IELT) and promote satisfaction level, but ideal dosing remains to be determined. Tramadol is well tolerated at a dose of 25 to 50 mg [10,11].

Therefore, we conducted this randomized controlled trial to compare the efficacy of tramadol and paroxetine in the treatment of primary PE.

## Materials and methods

This randomized controlled trial was conducted at the outpatient department of Nishtar Hospital, Multan, from January 2017 to January 2018. All patients were diagnosed based on history, physical examination, and a dedicated validated questionnaire. Proper approval was obtained from the ethics committee and institutional review board. Potent married men aged 30 to 40 years who had been in a relationship for at least six months with uncontrolled ejaculation within one minute of vaginal intromission were included in the study. Patients with low libido, erectile dysfunction, psychiatric disorders, or psychological conditions such as depression, alcohol abuse, use of sedative drugs, or having organic illnesses like hyperthyroidism, asthma, hypothyroidism, diabetes, and cardiac arrhythmias which would limit the use of SSRIs were excluded from the study. Patients with any history of previous use of treatments for PE within the three months from the presentation were excluded as well. IELT was defined as the time from vaginal intromission to intravaginal ejaculation. The confidence interval was set at 95%, and the power of the study at 80%. P1 (i.e., control over ejaculation in the tramadol group from baseline to post-treatment) was 8.4%. P2 (i.e., control over ejaculation in the tramadol group from baseline to post-treatment) was 22%. A total of 106 patients were included in this study. Fifty-three patients were enrolled in the tramadol group and 53 in the paroxetine group.

Patients who fulfilled the inclusion criteria were randomly assigned into two groups: Group A and Group B.

Patients in Group A received 50 mg of tramadol two hours before planned intercourse. Group B patients received 20 mg of paroxetine four hours before planned intercourse once a day, thrice weekly for eight weeks. Informed consent was obtained before inclusion in the study. All patients were monitored via follow-up for eight weeks from the start of treatment on weekly basis.

Data were entered and analyzed using IBM SPSS Statistics for Windows, Version 23.0 (Armonk, NY: IBM Corp.). Mean and standard deviation, as well as frequency and percentages, were calculated for numerical data like age, duration of disease, gender, socioeconomic status, and IELT (IELT was noted on a stopwatch). A p-value ≤0.05 was considered statistically significant.

## Results

A total of 106 patients were included in the study; 53 patients were randomized to the tramadol group and 53 to the paroxetine group. The mean age of patients in the tramadol group was 31.8±3.8 years, and the mean age of patients in the paroxetine group was 32.2±4.1 years, respectively. The mean baseline IELT for patients in the tramadol group versus paroxetine group was 44.93±3.95 seconds and 43.74±2.14 seconds, respectively, with no statistically significant difference (p=0.057). The mean treatment IELT for patients in the tramadol and paroxetine groups was 136.91±5.61 seconds versus 95.42±5.53 seconds, respectively, denoting a statistically significant difference (p=0.000). The differences were statistically significant within the groups' baseline and treatment IELT (p<0.001; Table 1).

**Table 1:** Intravaginal ejaculatory latency time in both groups

Variables	Tramadol n=53	Paroxetine n=53	P-value
Age (years)	31.8±3.8	32.2±4.1	0.426
Baseline IELT (seconds)	44.93±3.95	43.74±2.14	0.057
Treatment IELT (seconds)	136.91±5.61	95.42±5.53	0.000
Paired sample test p-value	0.000	0.000	--

The mean baseline PE score of patients in the tramadol and paroxetine groups was 9.68±2.23 versus 9.15±1.05, respectively, with no significant difference (p=0.117). Ejaculation control, difficulty in ejaculation, and distress due to ejaculation in the tramadol group were noted in 26 (49.1%), 24 (45.3%), 2 (3.8%), and 9 (17%) patients, respectively. Ejaculation control, difficulty in ejaculation, and distress due to ejaculation in the paroxetine group were noted 33 (62.3%), 20 (37.7%), 7 (13.2%), and 20 (37.7%) patients respectively.

Meanwhile, the mean post-treatment PE score in the tramadol and paroxetine groups was 14.01±1.31 versus 9.82±1.96, respectively, a statistically significant difference (p=0.000). Ejaculation control, difficulty in ejaculation, and distress due to ejaculation in the tramadol group were noted in 13 (24.5%), 4 (7.5%), and 4 (7.5%) patients, respectively. Ejaculation control, difficulty in ejaculation, and distress due to ejaculation in the paroxetine group were noted in 26 (49.1%), 9 (17%), and 13 (24.5%), respectively. The differences were statistically significant between the groups' baseline and post-treatment PE (p<0.001; Table 2).

**Table 2:** Premature ejaculation profile score in both groups

Variables	Tramadol n=53	Paroxetine n=53	P-value
Baseline PE	9.68±2.23	9.15±1.05	0.117
Treatment PE	14.01±1.31	9.82±1.96	0.000
Paired sample test p-value	0.000	0.036	--
Baseline satisfaction with sexual intercourse			
Control over ejaculation	n=26 (49.1%)	n=33 (62.3%)	0.171
Difficulty	n=2 (3.8%)	n=7 (13.2%)	0.081
Ejaculation-related distress	n=9 (17%)	n=20 (37.7%)	0.017
After treatment satisfaction with sexual intercourse			
Control over ejaculation	n=13 (24.5%)	n=26 (49.1%)	0.009
Difficulty	n=4 (7.5%)	n=9 (17%)	0.139
Ejaculation-related distress	n=4 (7.5%)	n=13 (24.5%)	0.017

## Discussion

In our study, we observed a marked improvement in PE with the use of tramadol compared to paroxetine. A similar study was conducted by Hamidi-Madani et al., reporting that tramadol is an effective and safe alternative to paroxetine for the treatment of PE [12]. That study included three groups of patients who received tramadol, paroxetine, or placebo.** **A similar study was conducted by Zhang et al. and concluded that paroxetine is more effective and gave long-lasting results when used for the purpose of PE, but paroxetine in combination is more effective [13].

A study conducted by Wu et al. concluded that tramadol and paroxetine had no significant difference in terms of mean IELT; it also suggested that tramadol can be used as a treatment option for PE [14]. Safarinejad and Hosseini reported that tramadol can be used as PE treatment option and that it can increase the IELT when used for six weeks or more [15]. Both studies have findings that are consistent with ours.

Another study conducted by Kaynar et al. reported that similar findings regarding the use of tramadol [16]. Compared to placebo, after eight weeks of treatment of PE with tramadol, a significant increase in mean IELT was noted. In another study by Alghobary et al., controversial results were seen that at sixth-week treatment both paroxetine and tramadol increase the IELT 11 fold and seven fold respectively, but at 12-week treatment, tramadol decreases the IELT by five fold but paroxetine increases it 22 folds [17]. 

Bar-Or et al. compared two different doses of tramadol (62 mg and 89 mg) versus placebo for 12 weeks and observed an increase in IELT in all groups, with tramadol 62 mg being more effective, given that a 4.2-fold significant increase from baseline was observed [18]. Ozcan et al. reported controversial findings suggesting that paroxetine increased all four indicators of PE [19].

Patrick et al. reported increase in all four items of PE when tramadol was used as an alternative to all other medications [20]. Salem et al. reported that "on demand" use of tramadol for six weeks increased mean IELT [21]. Collectively, these results allow medical practitioners to consider the use of tramadol for PE treatment.

Limitations

Assessment of all variables was dependent on self-estimation of patient using stopwatch which has some degree of bias regarding the exact time. Further studies are needed to collect observations with some strong and more reliable methods.

## Conclusions

Tramadol is effective and useful compared to paroxetine in the treatment of PE. Tramadol can be used as an alternative to other medications for the treatment of lifelong PE.
